# An atypical receiver domain controls the dynamic polar localization of the *Myxococcus xanthus* social motility protein FrzS

**DOI:** 10.1111/j.1365-2958.2007.05785.x

**Published:** 2007-07-01

**Authors:** James S Fraser, John P Merlie, Nathaniel Echols, Shellie R Weisfield, Tâm Mignot, David E Wemmer, David R Zusman, Tom Alber

**Affiliations:** 1 Department of Molecular and Cell Biology, University of California Berkeley, CA 94720-3320, USA.; 2 Department of Chemistry, University of California and Physical Biosciences Division, Lawrence Berkeley National Laboratory Berkeley, CA 94720-1460, USA.

## Abstract

The *Myxococcus xanthus* FrzS protein transits from pole-to-pole within the cell, accumulating at the pole that defines the direction of movement in social (S) motility. Here we show using atomic-resolution crystallography and NMR that the FrzS receiver domain (RD) displays the conserved switch Tyr102 in an unusual conformation, lacks the conserved Asp phosphorylation site, and fails to bind Mg^2+^ or the phosphoryl analogue, Mg^2+^·BeF_3_. Mutation of Asp55, closest to the canonical site of RD phosphorylation, showed no motility phenotype *in vivo*, demonstrating that phosphorylation at this site is not necessary for domain function. In contrast, the Tyr102Ala and His92Phe substitutions on the canonical output face of the FrzS RD abolished S-motility *in vivo*. Single-cell fluorescence microscopy measurements revealed a striking mislocalization of these mutant FrzS proteins to the trailing cell pole *in vivo*. The crystal structures of the mutants suggested that the observed conformation of Tyr102 in the wild-type FrzS RD is not sufficient for function. These results support the model that FrzS contains a novel ‘pseudo-receiver domain’ whose function requires recognition of the RD output face but not Asp phosphorylation.

## Introduction

Controlling movement is a central problem in bacterial physiology that depends, in many systems, on polar localization and activation of a few key proteins. The social (S) motility of species such as *Myxococcus xanthus* and twitching motility of *Pseudomonas aeruginosa* are powered by the polar retraction of polar type IV pili (TFP) (Sun *et al*., 1999; Wall and Kaiser, 1999; Mignot *et al*., 2005). The direction of movement is determined by the frequency of reversals along the long axis of the cell. Genetic studies in *M. xanthus* have identified the Frz signal transduction pathway as the source of the directional signal, but the mechanisms of environmental sensing, signalling and directional reversal are not well understood.

Social motility requires the FrzS protein (Ward *et al*., 2000). Remarkably, functional fusions of FrzS to green fluorescent protein (GFP) localize to the leading, piliated pole of the cell and oscillate between the leading and lagging poles (Mignot *et al*., 2005). Cell reversal correlates with FrzS accumulation at the new leading pole, and this localization pattern is itself essential for *M. xanthus* S-motility (Mignot *et al*., 2007b). The redistribution of FrzS from pole to pole depends on the activity of the Frz pathway, but the roles of FrzS in TFP-dependent motility and the biochemical mechanisms that underlie the polar release and transport of FrzS are unknown.

The domain structure of FrzS provides clues to its mechanism of action and localization. The N-terminal receiver domain (RD), a 258-residue coiled-coil domain, and the extreme C-terminal tail of the protein are all required for the dynamic subcellular localization and S-motility activity of FrzS. We hypothesized that the coiled-coil domain, via interaction with a putative cytoskeletal motor, serves to mediate FrzS movement between the cell poles while the RD and C-terminal tail function, respectively, as leading and lagging pole retention motifs (Mignot *et al*., 2005). In this study, we investigated the structure and function of the FrzS RD to determine if its role in FrzS localization depends, like canonical RDs, on a phosphorylation-based conformational change at the signal output face of the domain.

The mechanisms of RD function and signal transduction have been well characterized for several canonical RDs, such as the *Escherichia coli* response regulator CheY. Through three major functional elements, phosphorylation shifts the equilibrium population of CheY and other RD to favour the active conformation that binds the flagellar motor (Djordjevic and Stock, 1998; Silversmith and Bourret, 1999). First, the Asp57 phosphorylation site of CheY is part of a conserved acidic triad, including Asp12 and Asp13, that chelates the Mg^2+^ necessary for aspartic acid phosphorylation (Djordjevic and Stock, 1998). Second, the protein phosphorylation signal is transduced to the opposite side of the protein by a shift in the hydrogen-bonding network involving Lys109 and Thr87 (Schuster *et al*., 2001). The importance of these residues in aspartic acid phosphorylation is underscored by the positional conservation of the acidic triad, threonine and lysine residues in the active site of a phosphoserine phosphatase, a protein that contains no sequence similarity to RDs (Cho *et al*., 2001). Third, CheY phosphorylation ultimately results in the burial of the conserved Tyr106, which occludes the α4-β5-α5 face of the protein in the unphosphorylated ‘off’ state (Zhu *et al*., 1996; Lee *et al*., 2001). The burial of this ‘switch’ Tyr residue activates the RD by increasing affinity for downstream target proteins (McEvoy *et al*., 1999; Guhaniyogi *et al*., 2006).

Most canonical RDs found in multidomain proteins contain all three functional elements and convert phosphorylation into a switch in the conformation of the α4-β5-α5 face and the conserved aromatic residue corresponding to Tyr106 in CheY, which alters the protein interactions of the RD (Doucleff *et al*., 2005; Nixon *et al*., 2005). Recently, several proteins, such as the cyanobacterial circadian clock protein KaiA, have been characterized that contain RD-like structural folds that lack critical residues involved in aspartic acid phosphorylation and signal transduction (Williams *et al*., 2002). The considerable sequence divergence of these ‘pseudo-receiver’ domains suggests that they do not possess the signal input or output functions of canonical RDs.

Here we investigated the structure and the functional properties of the FrzS RD to determine if it responds to aspartate phosphorylation and propagates a signal in a manner analogous to canonical RDs, such as *E. coli* CheY. Surprisingly, despite sequence and structural similarity to canonical RDs, the FrzS RD did not bind Mg^2+^ or Mg·BeF_3_, lacked conserved residues that normally transduce a phosphorylation signal and did not require aspartic acid phosphorylation *in vivo*. In contrast, the switch Tyr and a neighbouring His residue were essential for S-motility and proper FrzS localization to the leading cell pole *in vivo*. Our data suggest that the FrzS RD mediates S-motility by a novel mechanism that requires the canonical signal output face but does not involve aspartic-acid phosphorylation.

## Results

### FrzS RD has unusual sequence features

The N-terminal 124 amino acids of FrzS are significantly similar to canonical RDs, as judged using blast and pfam-hmm (Bateman *et al*., 2004). In a phylogenetic analysis of canonical RDs and the cyanobacterial pseudo-RD KaiA, FrzS is part of a distinct clade within canonical RDs (Supplemental Fig. S1). KaiA, however, clusters with other pseudo-RDs as an outgroup (Supplemental Fig. S1).

**Fig. 1:**
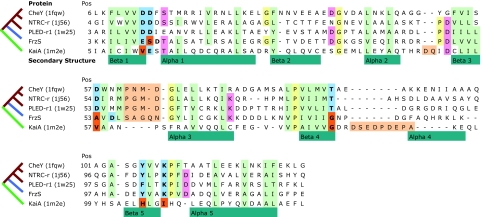
The FrzS RD sequence differs from canonical RDs and pseudo-RDs. Sequence alignment of representative canonical RDs, FrzS RD and KaiA pseudo-RD. The schematic tree shown at the left is representative of the sequence groupings (red = canonical-RD, blue = FrzS-group-RD, green = pseudo-RD), but branch lengths differ from the quantitative tree (Supplemental Fig. S1). Sequence alignment was generated using the MUSTANG alignment algorithm (Konagurthu *et al*., 2006). Regions of poor structural alignment delineating KaiA from canonical receivers and FrzS are indicated in light orange. Important RD sites are shown in blue, with deviations from canonical residues in KaiA and FrzS shown in dark orange. Conserved hydrophobic residues are highlighted in light green, conserved polar and charged residues in pink, and conserved prolines and glycines in yellow. The secondary structure of FrzS is indicated underneath the alignment.

Examining the sequence of FrzS revealed an unusual pattern of substitutions compared with canonical RDs (Fig. 1). FrzS Tyr102 corresponds to the conserved conformational switch residue on the α4-β5-α5 output face of the protein. However, several differences were observed in the area aligned to the phosphorylation site of canonical RDs. Ser10 occurs at the site of a highly conserved Asp residue that functions to bind Mg^2+^ to promote phosphorylation and dephosphorylation. Substitution of this residue in CheY (Asp13) greatly weakens the affinity of the protein for Mg^2+^, blocks phosphorylation and inactivates the switch *in vivo* (Bourret *et al*., 1990). In addition, it is difficult to align the phosphorylated Asp of canonical RDs to any Asp in FrzS. Residues that transduce the phosphorylation event to the switch tyrosine in CheY are altered in FrzS. Of the critical lysine and threonine residues involved in communication from the phosphorylation site to the α4-β5-α5 face, only the lysine is conserved. The threonine, which makes critical side chain hydrogen bonds in CheY, is substituted by a glycine in FrzS. These sequence patterns, which suggest that the FrzS RD has diverged from canonical response-regulator RDs, led us to investigate these unusual sequence features in their structural context.

### Structures of the FrzS RD

**Fig. 2:**
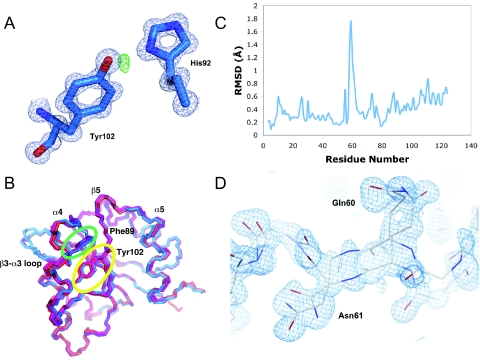
Crystal structure of the FrzS RD A. 2F_o_-F_c_ electron density (1 σ– light blue) and *F*_o_-*F*_c_ difference electron density (2 σ– green) at 1.0 Å resolution showing the putative switch Tyr102 and the neighbouring His92. Difference electron density indicates that there is a hydrogen bond between His92-Nδ1 and Tyr102-O. B. Overlay of the main chain of the FrzS RD in the hexagonal form crystals (blue) with three independent chains of the tetragonal form (red, magenta, purple) shows a high degree of similarity between independent structures of the FrzS RD. There are two conformers of the β3–α3 loop in the hexagonal form, one of which corresponds to the conformation seen in all three tetragonal monomers. The switch Tyr102 is in the same rotamer in all four chains and highlighted by a yellow circle. The position of Phe89, which renders the canonical ‘inward facing’ Tyr102 rotamer inaccessible, is highlighted by a green circle. C. Plot of main chain RMSD (Å) between the four independent FrzS RD chains highlights the variability in the β3-α3 loop and the similarities elsewhere in the structure. D. Multiple conformations of the β3-α3 loop seen in the 2F_o_-F_c_ electron density (1.5 σ– light blue) at 1.0 Å resolution in the hexagonal form of the FrzS RD reveal coordinated motions of this loop region.

To explore the potential for intramolecular signalling, we determined the crystal structure of the FrzS RD in two crystal forms (Table 1, Fig. 2), a hexagonal form (P6_3_) at 1.0 Å resolution and a tetragonal form (I4) at 1.9 Å. The hexagonal form was solved by molecular replacement using the first RD from the diguanylate cyclase PleD (Chan *et al*., 2004) as the search model. The R/R-free values for all data to 1.0 Å resolution were 9.2/12.3% (Table 1), below the average for structures at comparable resolution in the PDB. After several rounds of refinement using data to 1.0 Å resolution, this structure was used to solve the tetragonal form. The FrzS RD structures in the two crystal forms were very similar (Fig. 2B), but the overall Cα RMSD for the structures of 0.57 Å was above the errors in the coordinates (Fig. 2C). The largest differences between the structures in the two crystal forms occurred in the conformation of the β3-α3 loop, part of which was modelled in two conformations in the hexagonal structure (Fig. 2D). Excluding these residues (54–62) from the comparison reduced the Cα RMSD to 0.45 Å.

**Table 1:** Crystallographic data collection and refinement statistics.

	WT	WT	Tyr102Ala	His92Phe
Space group	P6_3_	I4	P6_3_	P2_1_
Unit cell (Å)	a = b = 64.4, c = 46.0	a = b = 142.5, c = 37.4	a = b = 63.8, c = 46.7	a = 34.9, b = 37.1, c = 42.1, β = 102.5°
Wavelength (Å)	0.8865	1.15	1.15	0.8856
Resolution (Å)	20–1.0	20–1.9	20–1.3	20–1.02
Reflections	58 872 (5 841)	30 067 (4 230)	26 743 (3 851)	51 778 (6 353)
Mean I/σ(I)	28.75 (2.5)	6.8 (1.6)	12.0 (2.7)	14.3 (2.0)
Completeness (%)	100 (100)	98.7 (97.9)	100 (100)	96.7 (82.4)
Redundancy	8.5 (6.7)	1.9 (1.7)	7.7 (7.4)	3.5 (2.2)
Rsym (%)	6.1 (45.0)	7.6 (64.0)	9.3 (83.7)	4.3 (32.7)
Wilson B	6.9	25.0	12.1	6.5
Rcryst (%)	9.2	22.0	13.2	12.3
R-free (%)	12.3	25.4	16.2	15.4
Protein atoms^a^	1 138	2 687	972	1 083
Solvent atoms	362	266	211	246
Average B	14.9	28.5	17.2	12.2
RMS Δ angles (°)	1.94	0.826^b^	1.59	1.54
RMS Δ bonds (Å)	0.014	0.005^b^	0.015	0.012
PDB ID	2gkg	2i6f	2nt3	2nt4

Values in parentheses refer to the outermost resolution shell.

a Atom counts exclude alternate conformations.

b Partial disorder of the third chain of the tetragonal crystal form necessitated very tight geometric restraints; as a result, values for deviations from ideal geometry are lower than average.

The tetragonal crystals contained three molecules in the asymmetric unit, one of which forms one side of a large solvent channel in the crystal lattice. Although the backbone conformation was very similar, this molecule was poorly ordered in the present structure, and virtually missing from the electron density when different cryoprotectants were used (N. Echols and J. Fraser, data not shown). Due to the lower resolution and disorder of this structure, we have focused our analysis on the hexagonal form. Nonetheless, the independent structures in the tetragonal form provide an important control for the effects of crystal packing on the overall conformation. Even in the relatively unconstrained tetragonal crystal form, FrzS adopted approximately the same structure observed in the high-resolution crystals. The crystal contacts along the α4-β5-α5 face varied in the four independent molecules in the two crystal forms, suggesting that the similar side chain positions of critical residues, including Tyr102, faithfully represent the predominant conformation of the RD.

### Comparison to CheY

**Fig. 3:**
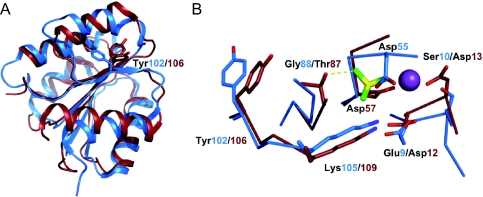
Comparison of the FrzS RD and CheY structures A. FrzS and CheY have similar global structures, but the FrzS ‘switch’ Tyr102 is in a different position in the two RDs. Ribbon representation showing FrzS (blue) and meta-active CheY (1jbe, red). The two conformations representing inactive and active states of CheY Tyr106 differ from the distinct rotamer of FrzS Tyr102 (sticks). B. Superimposed FrzS (blue) and phosphorylated CheY (1fqw, red) contain the switch Tyr pointed into distinct pockets in the respective proteins. The FrzS structure reveals that this RD does not support Asp phosphorylation or allosteric coupling to the Asp triad. FrzS Ala53 replaces the CheY Asp57 phosphorylation site, and the FrzS Asp55 Cα is shifted > 5 Å relative to CheY Asp57. FrzS Ser10 replaces CheY Asp13, which is required for Mg^2+^ binding in CheY. FrzS also lacks the Thr87 side chain required to functionally link the Asp57 phosphorylation to Tyr106 in CheY. Residues are shown in sticks labelled according to their identities in FrzS/CheY. BeF_3_ is shown as yellow/green sticks and Mg^2+^ is shown as a purple sphere.

The structure of the FrzS RD aligns well with the first RD of *Caulobacter crescentus* PleD (Cα RMSD = 1.5 Å, sequence identity = 27%) and both the phosphorylated and unphosphorylated forms of *Eschericia coli* CheY (Cα RMSD = 1.5 Å (phosphorylated)/1.5 Å (unphosphorylated), sequence identity = 19%). Despite the overall similarity of the fold (Fig. 3A), several structural features distinguish the FrzS RD from these conventional RDs. The N-termini of helix α2 (residues 35–45) and helix α4 (residues 86–94) were conformationally strained in FrzS, and the latter was identified by DSSP as a 3_10_ helix. This feature also was clearly observable in the electron density for all three monomers in the tetragonal form.

In canonical RDs, the rotameric state of an aromatic residue on β5 correlates with the signalling state of the RD (Lee *et al*., 2001). In the FrzS RD, the conserved Tyr102 adopted an ‘inward-facing’ rotamer. Remarkably, this conformation was distinct from those in either the inactive or active CheY (Fig. 3A). Instead, the Tyr102 rotamer in FrzS was identical to that of the equivalent Phe102 in the first RD of PleD, where it was identified as an inactive conformation (Chan *et al*., 2004). The conformation of the β4-α4 loop and the packing of Phe89 against Tyr102 suggest that the canonical active rotamer is inaccessible (Fig. 2B). Furthermore, the conformation of Tyr102 is stabilized by hydrogen bonding of the hydroxyl group to His92 Nδ1, clearly visible in difference electron density in the high-resolution structure (Fig. 2A). This network has analogies to the conserved hydrogen bond of the switch tyrosine to a backbone amide in helix α4 in other RDs.

A previous 1.1 Å-resolution structure of CheY showed sampling of both the ‘on’ and ‘off’ conformations in the absence of phosphorylation (Simonovic and Volz, 2001). Similarly, NMR data show that RDs dynamically sample both signalling states in the absence of phosphorylation (Volkman *et al*., 2001). To search for this underlying dynamic switch in the FrzS RD, we examined the crystal structures for structural polymorphism in the switching network. In contrast to the CheY structure, we observed no conformational heterogeneity along the α4-β5-α5 face of the 1.0 Å FrzS RD structure, even at low contours of electron density below 0.25 σ. The β3-α3 loop showed the highest degree of variability between the four independent monomers of FrzS. In the high-resolution hexagonal structure, a discrete minor population was modelled at 34% occupancy for the side-chain and backbone atoms of residues 55–62 (Fig. 2D). This alternate conformation suggests a coordinated mode of motion, but this segment has not been implicated in dynamic rearrangements associated with RD signalling.

Comparing residues in the FrzS RD to those involved in the allosteric communication between the phosphorylation site and the output α4-β5-α5 face of CheY showed further structural differences between the two domains (Fig. 3B). In the acidic binding pocket of FrzS, Ser10 replaces the essential conserved Asp13 in CheY, and Ala53 in FrzS occurs in the position of the phosphorylated Asp57 in CheY. The closest Asp in FrzS was Asp55, which occurs two residues out of the β3-α3 loop. Although the Cα atoms of these Asp residues were 5.4 Å apart in the superimposed structures, the side chain carboxyl group of FrzS Asp55 was 1.8 Å from the position of the CheY Asp57 side chain. Although FrzS Lys105 aligns well with CheY Lys109, the other side chain that plays a key role in transducing the phosphorylation to the output face, Thr87, is substituted by a glycine (Gly88) in FrzS. The position of Tyr102 in FrzS is partially buried, suggesting that apo-FrzS does not prevent interactions along the α4-β5-α5 face by steric clashes with the switch tyrosine. In this respect, apo-FrzS is structurally similar to phosphorylated CheY. In summary, comparison of the RD structures revealed that FrzS lacks strict analogues of two of the three conserved acidic residues that mediate RD Asp phosphorylation and contains a Gly substitution for a residue essential to transduce the phosphorylation signal to the output face in CheY. These changes at essential signalling sites raised the possibility that the FrzS RD is not Asp-phosphorylated.

### The FrzS RD is insensitive to Mg^*2*+^ and Mg^*2*+^·BeF_3_

**Fig. 4:**
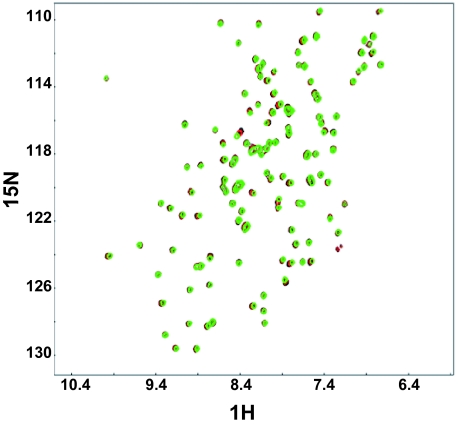
Mg^2+^ does not bind the FrzS RD. ^15^N-HSQC spectra of the FrzS RD in the presence of 0 mM (black), 30 mM (red) and 60 mM (green) MgCl_2_. Chemical shifts of the backbone amide peaks showed no changes associated with Mg^2+^ addition. This insensitivity suggested that FrzS failed to bind Mg^2+^, which is a signature of competence for Asp phosphorylation of canonical RDs.

To test this idea, we used NMR experiments to determine whether the FrzS RD bound Mg^2+^ or Mg^2+^·BeF_3_, as seen previously for RDs that respond to Asp phosphorylation (Yan *et al*., 1999). In ^15^N-HSQC spectra (Fig. 4), peaks for the amide hydrogens were relatively sharp and well dispersed, indicating a folded structure. Because chemical shift is an exquisitely sensitive measure of the surrounding environment, the ^15^N-HSQC spectra of RDs typically change upon addition of the physiological cofactor, Mg^2+^. We titrated in Mg^2+^ (for which RDs typically have a K_d_ in the mM range) up to 100 mM and detected no significant changes in chemical shifts (Fig. 4). We also titrated in Mg^2+^ and BeF_3_, which mimics phosphorylation on aspartic acid in RD active sites (Yan *et al*., 1999; Wemmer and Kern, 2005), and noted no changes (J. Fraser and D. Wemmer, data not shown). These data provide strong evidence that the FrzS RD does not bind Mg^2+^ or Mg^2+^·BeF_3_. This conclusion implies that, unlike canonical RDs, the FrzS RD is not Asp-phosphorylated.

### S-Motility does not require FrzS RD Asp phosphorylation

To explore the requirements for FrzS RD function, we engineered mutations of the putative phosphorylation site and switching network and determined their phenotypes when introduced into *M. xanthus*. For these experiments, we began with a strain that expresses wild-type FrzS protein fused at its C-terminus to the green fluorescent protein (FrzS-GFP). This fusion protein supports essentially wild-type levels of S-motility and enables characterization of mutational effects on the subcellular localization of FrzS (Mignot *et al*., 2005). The FrzS coding sequence of this fusion gene was mutated to produce FrzS-GFP carrying the desired amino acid changes in the RD.

**Fig. 5:**
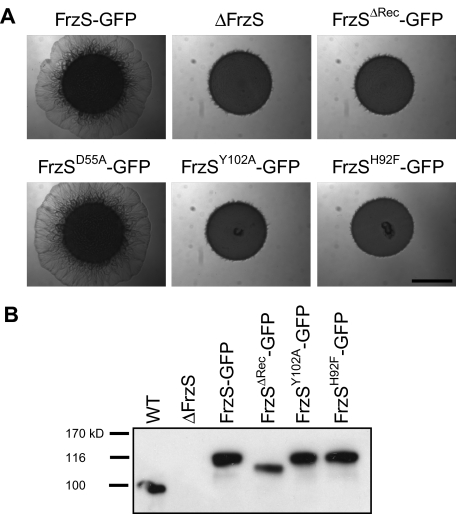
FrzS mutations Tyr102Ala and His92Phe, but not Asp55Ala, abolished S-motility *in vivo* A. *Myxococcus xanthus* S-motility phenotypes of FrzS RD point mutants. FrzS-GFP (TM3), ΔFrzS (DZ4526), FrzS^ΔRec–GFP^ (DZ4535), FrzS^Asp55Ala^ (DZ4539), FrzS^Tyr102Ala^ (DZ4538) and FrzS^His92Phe^ (DZ4550) were spotted at 4 × 10^7^ cells ml^−1^ on nutrient rich CYE media containing 0.5% agar and incubated for 24 h at 32°C. Scale bar equals 5 mm. The Asp55Ala FrzS mutant supported wild-type levels of S-motility, while the phenotypes of the Tyr102Ala and His92Phe FrzS mutants were indistinguishable from the deletions of the RD or all of FrzS. B. Anti-FrzS Western blot of whole cell lysates of wild-type *M. xanthus* and the strains shown in A. DZ4539 is not shown. The Asp55Ala, His92Phe and Tyr102Ala FrzS variants were expressed at equivalent levels *in vivo*.

We first tested the functional requirement for Asp55, the aspartate residue in FrzS closest to the normal site of phosphorylation in canonical RDs. We mutated this residue to an alanine in FrzS-GFP, and in a standard soft agar colony spreading assay, we observed no defect in the S-motility of an *M. xanthus* strain expressing the Asp55Ala FrzS-GFP variant (Fig. 5A). Colony size and morphology in the Asp55Ala mutant were indistinguishable from the wild-type, GFP-fusion strain. These results suggest that Asp55 phosphorylation is not essential for FrzS function *in vivo.*

### Social motility and the α4-β5-α5 output face

Although the RD of FrzS is unlikely to undergo a canonical phosphorylation event, the output face of the RD may still regulate the activity of the protein in S-motility. This hypothesis is supported by the conservation of Tyr102, a residue that in canonical RDs is located on the output face of the molecule and governs interactions with downstream effector domains. We tested the functional importance of Tyr102 by mutating this residue to an alanine in FrzS-GFP and examining the social motility phenotype of the resulting strain. *M. xanthus* expressing FrzS-GFP Tyr102Ala failed to undergo normal colony expansion in the S-motility assay. As judged by colony morphology, the Tyr102Ala mutation created a S-motility defect as severe as that seen for a deletion of the entire FrzS RD, FrzS^ΔRec^-GFP, or for a deletion of the entire FrzS protein, ΔFrzS (Fig. 5A). Western blotting analysis showed equal levels of FrzS-GFP Tyr102Ala and wild-type FrzS-GFP *in vivo* (Fig. 5B), indicating that the phenotype of the Frz Tyr102Ala variant was not the result of decreased levels of the mutant protein.

To test whether the environment of Tyr102 is important for domain function, we mutated His92, which forms a hydrogen bond to the buried Tyr102 hydroxyl group in the FrzS RD structure, to a phenylalanine. The His92Phe mutation blocked social motility to the same extent as the Tyr102Ala mutation with no effect on protein stability (Fig. 5A and B). Therefore, both Tyr102 and His92, a direct contact residue buried under Tyr102 are essential for FrzS function in S-motility.

### Tyr102 and His92 are required for the leading-pole localization of FrzS

**Fig. 6:**
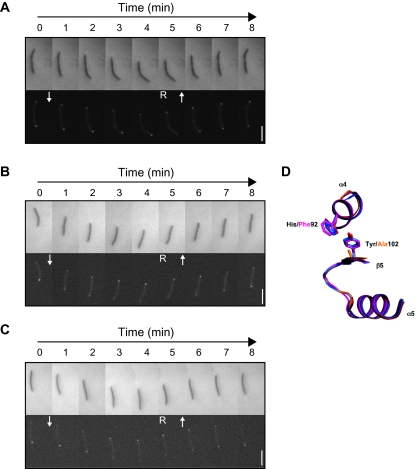
Subcellular localization of FrzS-GFP variants during *Myxococcus xanthus* movement correlates with S-motility phenotypes and crystal structures of FrzS RD variants. A. Time-lapse image sequences showing the cell location (top) and GFP fluorescence signal (bottom). Arrows indicate the direction of cell movement. ‘R’ indicates the frame in which a cell reversal took place. Scale bar = 5 μm. B. Time-lapse image sequence showing the subcellular localization of FrzS^Tyr102Ala^-GFP during *Myxococcus xanthus* movement. The mutant FrzS was localized at the lagging pole, rather than the leading pole. Scale bar = 5 μm. C. Time-lapse image sequence showing the subcellular localization of FrzS^His92Phe^-GFP during *Myxococcus xanthus* movement. The localization pattern of His92Phe FrzS matched that of the Tyr102Ala mutant, indicating that both of these RD residues are required for normal FrzS localization. Scale bar = 5 μm. D. The structures of RD mutants Tyr102Ala (orange) and His92Phe (magenta) are globally similar along the α4-β5-α5 output face to the structure of WT FrzS (blue). This similarity suggests the mutant and wild-type structures are in the same signalling state, and that the buried nature of the Tyr102 rotamer is critical in switching the signalling or binding state of FrzS.

The severe defect in S-motility produced by the Tyr102Ala and His92Phe mutations prompted us to characterize their effects on the subcellular localization of FrzS-GFP. Using a agar slab motility assay we determined the localization dynamics of FrzS-GFP Tyr102Ala (or His92Phe) during *M. xanthus* cell motility (Fig. 6A and Supplemental Fig. S2A). In wild-type cells, FrzS-GFP is localized to the leading pole of the cell during forward movement ((Mignot *et al*., 2005) and Fig. 6A. Small amounts of FrzS-GFP are gradually redistributed to the lagging pole as cell movement continues. Coincident with cell reversal, the bulk of the remaining FrzS-GFP redistributes to the lagging pole. After cell reversal the bulk of FrzS-GFP is stably retained at the new leading pole while small amounts of the protein again slowly redistribute to the new lagging pole.

We observed a significantly different pattern of subcellular localization in cells containing Tyr102Ala FrzS-GFP. As these cells move forward over the agar surface, Tyr102Ala FrzS-GFP localizes primarily to the lagging cell pole in a manner highly reminiscent of FrzS^ΔRec^-GFP (Fig. 6B and Supplemental Fig. S2B). Upon cell reversal, the bulk of FrzS-GFP Tyr102Ala rapidly (within 1–2 min) redistributes from the site of the new leading pole to the new lagging cell pole. The same localization pattern was observed for His92Phe FrzS-GFP (Fig. 6C and Supplemental Fig. S2C). These results suggest that Tyr102 and the surrounding region are essential for the N-terminal RD to stabilize FrzS localization at the leading cell pole and confirms the requirement of this localization pattern for *M. xanthus* social motility.

### Structures of Tyr102Ala and His92Phe FrzS variants

Because the Tyr102Ala and His92Phe FrzS mutations caused defects in S-motility and leading pole localization (Figs 5 and 6), we sought to understand the structural basis for these effects. Atomic resolution crystal structures of both mutants showed a similar backbone structure compared with the wild-type FrzS RD (Table 1, Fig. 6D). The Tyr102Ala mutant crystallized in similar conditions and in the same hexagonal space group as the high-resolution wild-type form. The His92Phe mutant crystallized in a distinct monoclinic space group (P2_1_), with one crystal contact within 4 Å of the α4-β5-α5 output face, as quantified by the Crystal Contacts Analysis Server (Eyal *et al*., 2005). In comparison to the α4-β5-α5 face in the hexagonal crystals (wild type and Tyr102Ala variants), only Asp118 had a higher contact area in the His92Phe FrzS RD. Nine crystal contacts on the α4-β5-α5 face observed in the hexagonal crystals were lost in the His92Phe RD crystals and one residue (Lys96) showed a reduced contact surface. In total, the intermolecular contact area decreased from an average of 4806 Å^2^/RD-monomer in the hexagonal crystals to 1594 Å^2^/monomer in the monoclinic His92Phe RD crystals. Although we cannot rule out potential allosteric effects due to distant interactions, the accessibility of the output face and the overall reduction in contact area suggest that the conformation adopted by the His92Phe FrzS RD, particularly at the site of the mutation, is not simply a result of crystallization.

The His92Phe replacement was expected to cause the Tyr102 side chain to switch to the outward facing rotamer due to the increased volume of Phe and the loss of the hydrogen bond between Tyr102 and the His92 side chains. Unexpectedly, Tyr102 remained facing inward in the structure of the His92Phe variant, and the Phe92 side chain shifted away from Tyr102 (Fig. 6D). Neither Phe92 nor the wild-type His92 are solvent accessible from the output face. The overall Cα RMSD for the His92Phe variant and hexagonal wild-type RD structures was 0.35 Å. The conformation of the β3-α3 loop in the mutant RD is nearly identical to the major conformation observed in the hexagonal wild-type RD structure. The structural similarity of the residues on the α4-β5-α5 face in the His92Phe RD and the wild-type protein also was quite striking (Fig. 6D). The all-atom RMSD was low, 0.53 Å, for this area (residues 86–120). In summary, the His92Phe RD structure is nearly identical to the hexagonal wild-type RD structure in both the dynamic β3-α3 loop and the output face of the RD.

Similarly, no large rearrangements were seen in response to the loss of side-chain volume in the Tyr102Ala mutant. The overall Cα RMSD for the Tyr102Ala and wild-type RD structures was 0.23 Å. In response to this mutation, His92 switched to a more common rotamer that does not present Nδ1 to residue 102 for hydrogen bonding. This feature was clearly observable in the atomic-resolution electron density. There were no major structural rearrangements along the α4-β5-α5 face (Fig. 6D), and the all-atom RMSD for this area was low (0.43 Å). The close similarity of the Tyr102Ala and wild-type RD structures coupled with the loss of S-motility of the mutant suggests that the Tyr102 side chain plays a direct role in FrzS function.

## Discussion

### Differences between regulation of canonical RDs and the FrzS RD

The combination of *in vivo*, crystallographic and NMR data suggest that the FrzS RD retains structural and functional features of canonical RDs, but does not modulate switching or signalling via aspartate phosphorylation. While the FrzS RD fold is highly similar to typical RDs, the FrzS RD structure lacked conserved features including the acidic triad, the Asp-phosphorylation site and a key residue implicated in intramolecular signalling. Titrations with Mg^2+^ and Mg^2+^·BeF_3_ monitored using NMR showed unambiguously that these ligands, which interact in the mM range with canonical RDs, failed to bind FrzS even at 100 mM. These NMR spectra are particularly sensitive to ligand binding that would change the electronic environment of residues near the binding site. The absence of changes in any FrzS RD nitrogen or proton chemical shifts even at high ligand concentrations differs from the behaviour of canonical RDs (Volkman *et al*., 2001; Wemmer and Kern, 2005) and provides strong evidence that Mg^2+^ and Mg^2+^·BeF_3_ do not bind. In turn, this conclusion implies that the Ser10-Asp13-Asp55 triad of the FrzS RD, unlike the analogous residues in canonical RDs, does not chelate Mg^2+^ or catalyse aspartyl-phosphate hydrolysis. Moreover, the lack of a side chain equivalent to Thr87 in CheY suggests that there is no functional communication between the Ser10-Asp13-Asp55 triad and the α4-β5-α5 face. In agreement with these indirect, *in vitro* results, the Asp55Ala FrzS mutation had no effect on S-motility *in vivo*. Taken together, these results imply that, in contrast to canonical RDs, the FrzS RD is not regulated by aspartate phosphorylation.

### FrzS function is altered by mutation along the α4-β5-α5 face

Despite these lines of evidence that the FrzS RD does not undergo the phosphorylation-based conformational switch typical of canonical RDs, residues along the α4-β5-α5 face of the molecule are essential for *M. xanthus* S-motility. The Tyr102Ala and His92Phe mutations, respectively, on or underlying the α4-β5-α5 face abolished S-motility but caused neither large changes in the RD crystal structure nor significant changes in FrzS protein levels *in vivo*. The structural similarities are unlikely to result from crystal packing forces, because four independent wild-type RD monomers displayed similar structures, and the His92Phe variant formed monoclinic crystals in which the α4-β4-α5 face was mostly free of lattice contacts. Because the RD itself is required for FrzS function in S-motility, these results suggest that the conformation of the output face of the domain plays a critical role in overall domain function (Mignot *et al*., 2005). The null phenotype of the Tyr102Ala mutant shows that this residue is essential for FrzS activity. The similarly inactivating His92Phe mutation, however, preserved the conformation of Tyr102 in the crystal structure of the mutant protein. This finding suggests that the conformation of Tyr102 shared by the wild-type and His92Phe variants is insufficient by itself to mediate function. In the observed structures, His92 and Phe92 are buried under the output face, out of position to make direct contacts with binding partners. Rather, the His92Phe mutation may compromise an alternative conformation of the α4-β5-α5 face or interrupt a signalling pathway required to switch the conformation of Tyr102 and to retain FrzS at the leading pole during S-motility.

In canonical RDs, the output face of the molecule acts as the focal point of signal output through interactions with target proteins (Djordjevic and Stock, 1998; Doucleff *et al*., 2005). By analogy, FrzS residues such as Tyr102 and His92 may mediate directly or indirectly interactions with target proteins. Here we monitored the localization of FrzS variants in single cells during S-motility in order to establish correlations between RD structure and function. The localization defects (Fig. 6) and null S-motility phenotypes of the His92Phe and Tyr102Ala variants support the idea that a physical interaction with the output face of the FrzS RD directly mediates the retention of FrzS at the leading cell pole. These results raise the possibility that a novel ‘backwards’ switch operates in the FrzS RD. The buried switch tyrosine of the unmodified (apo) FrzS RD is distinct from the exposed switch tyrosine in canonical RDs, which is buried upon Asp-phosphorylation (Fig. 3A). The burial of Tyr102 in the FrzS RD structure suggests the possibility that the apo-protein binds cognate effectors (i.e. the switch is ‘wired backwards’ compared with CheY). In this model, the apo-FrzS RD would bind targets along the α4-β5-α5 face and switch to a non-permissive exposed form in response to a signal. In an alternative form of this model, a distinct conformation with Tyr102 flipped out into the α4-β5-α5 face may bind cognate effectors.

Because we have not observed a conformational change in the FrzS RD, however, our data do not rule out the possibility that the output face affords a static binding surface that regulates FrzS localization but does not undergo a conformational change centred on Tyr102. In this unconventional model, binding and localization of FrzS would be regulated by conformational changes in partner proteins that contact Tyr102. This alternative model, however, does not provide a simple explanation for conservation of Tyr102 and other residues in the RD signal transduction network. In addition, the null phenotype of the His92Phe mutant, which does not alter the structure of the output face, implies that this mutation exerts an indirect effect on binding, presumably by altering the tendency of Tyr102 to undergo a classical rotameric switch. Because the FrzS RD does not fit the paradigm of regulation by Asp phosphorylation, it will be of interest to explore the nature of target-protein interactions that may reflect a new mode of RD regulation.

### Roles of RDs and pseudo-RDs in polar protein targeting

A variety of canonical RDs mediate protein targeting. For example, two well-characterized *C. crescentus* RD-containing proteins, DivK and PleD, localize to the cell poles. The PleD diguanylate cyclase concentrates at the cell pole upon phosphorylation of one of its two RDs (Chan *et al*., 2004). DivK, consisting of a single canonical RD, displays dynamic phosphorylation-dependent switching between cell poles with different biochemical compositions and functions (Lam *et al*., 2003; Matroule *et al*., 2004).

Pseudo-RDs are also capable of mediating polar protein localization. Several pseudo-RDs in the cyanobacterium *Synechococcus elongatus* PCC 7942 lack key residues required for phosphorylation (Williams *et al*., 2002; Mutsuda *et al*., 2003). One of these proteins, the histidine kinase CikA, has recently been shown to localize to the cell pole (Zhang *et al*., 2006). This localization depends on the CikA pseudo-RD. In its absence, CikA becomes diffusely localized throughout the cell. The protein FimX from *P. aeruginosa* provides an additional example of this phenomenon. FimX is a phosphodiesterase that (like FrzS) appears to play an essential role in TFP-based motility. FimX in wild-type cells is predominantly localized to a single cell pole and this localization pattern requires an N-terminal pseudo-RD (Kazmierczak *et al*., 2006). Pseudo-RDs (such as KaiA) lack the phosphorylation switch residues and contain extended loop regions that align poorly to canonical RDs. This sequence divergence from canonical RDs (Supplemental Fig. S1) reflects the lack of selection for residues that couple phosphorylation to alterations in protein–protein interactions. For this reason, these domains have been proposed to function as protein binding modules that are not regulated by phosphorylation (Kageyama *et al*., 2006). Furthermore, the lack of sequence conservation in the output face of pseudo-receivers suggests that they mediate polar localization by interaction mechanisms distinct from those operating in canonical, polar RDs.

### Implications for the mechanism of FrzS RD activity

How does the RD generate the dynamic leading pole localization pattern of the FrzS protein? In key functional residues, characteristic structural details, and response to mutations, the FrzS RD differs from canonical RDs. However, the FrzS RD is not as deeply diverged as other pseudo-RDs (Supplemental Fig. S1). In addition, the FrzS RD contains some of the conserved residues around the canonical phosphorylation pocket and the output face. We hypothesize that this domain in FrzS represents a novel ‘hybrid’ between canonical RDs and pseudo-RDs. In this model, the conserved output face would act in a manner reminiscent of canonical RDs to mediate stable retention at the leading cell pole. Polar release, however, would involve either a conformational change at the output face independent of RD phosphorylation or recognition of key conserved residues such as His92 and Tyr102. This pattern suggests that a distinct localization interface in FrzS that competes with the RD. Reversible regulation of one or both of these FrzS localization mechanisms likely accounts for the polar cycling of the protein in S-motility.

Atypical pseudo-RDs resembling FrzS appear to be common in the *M. xanthus* genome. In addition to FrzS, several other characterized proteins (such as AglZ and FrzG) and several uncharacterized *M. xanthus* proteins contain RDs missing residues essential for phosphorylation. AglZ, a protein that is essential for *M. xanthus* A-motility and has a domain structure similar to that of FrzS, contains an atypical RD with residues equivalent to FrzS His92 and Tyr102 (Mignot *et al*., 2007a). These sequence similarities suggests that the AglZ and FrzS RDs may perform analogous functions in different motility pathways. Immediate challenges are to determine how these atypical domains function and to understand how they interact with the canonical RD proteins in the signal transduction networks that govern the diverse behaviours of *M. xanthus* and other species. Our finding that the FrzS RD lacks the canonical Asp phosphorylation response yet maintains the essential nature of the α4-β5-α5 face may be generally applicable to these and other homologues.

## Experimental procedures

### Sequence analysis

Sequences similar to FrzS RD and KaiA pseudo-RD were collected by psi-blast and filtered, removing sequences that were 95% identical. The RD and pseudo-RD regions were aligned using Muscle (Edgar, 2004). An average distance tree using percent identity was calculated in Jalview (Clamp *et al*., 2004).

### Protein production

The RD of FrzS (1–124) was amplified from genomic DNA and ligated into the pET28b vector, which was transformed into BL21 + cells (Invitrogen). Protein was expressed by growing cells to an OD_600_ between 0.5 and 0.9 in Terrific Broth at 37°C and inducing expression with 1 mM isopropylthiogalactoside (IPTG) for 4 h at 37°C. Cells were centrifuged and flash-frozen in liquid nitrogen prior to lysis. The pellets were resuspended in 300 mM NaCl, 20 mM Tris pH 7.5, 0.5 mM Tris(2-carboxyethyl)phosphine hydrochloride (TCEP), 20 mM imidazole, and 5% glycerol and applied to an Ni-charged HiTrap Chelating HP column (GE Healthcare). Protein was eluted with resuspension buffer plus 250 mM imidazole and dialysed overnight into resuspension buffer in the presence of thrombin. The solution was passed through a second HiTrap Chelating column to remove the histidine tag and uncleaved protein, and the flow-through was collected and concentrated. The protein was further purified on a Superdex 75 column (GE Healthcare) equilibrated with 50 mM NaCl, 20 mM Tris pH 7.5 and 0.5 mM TCEP. Mutants were constructed using Quikchange kits (Stratagene) and purified using the same protocol.

### Crystallization and X-ray data collection

Purified FrzS RD variants at 20 mg ml^−1^ in 50 mM NaCl, 20 mM Tris pH 7.5, and 0.5 mM TCEP were mixed 1:1 with crystallization solution in microbatch trays and covered with Al's Oil (Hampton Research). Crystals for the RD were obtained in a wide variety of PEG solutions at multiple pHs and salt concentrations. Large crystals grew in as little as 2 days, but the best resolution was obtained using slower-growing crystals. Hexagonal crystals were grown in 0.1 M sodium formate and 23% PEG 3350; tetragonal crystals were grown in 0.2 M NaCl and 30% PEG 3350. Crystals were briefly soaked in well solution plus 10% xylitol and frozen in liquid nitrogen prior to data collection. The His92Phe mutant was grown by vapour diffusion in sitting drops with a well solution of 0.1 M BisTris pH 5.5 and 25% PEG 3350 and frozen in well solution plus 20% MPD. The Tyr102Ala variant was grown in microbatch trays with a well solution of 0.1 M MIB buffer, pH 7 (Newman *et al*., 2005) and 25% w/v PEG 1500, and frozen in well solution plus 30% PEG 400.

X-ray diffraction data were collected at ALS beamline 8.3.1 from a single crystal of each variant. For the high-resolution data sets, two separate wedges were combined, one with short exposures and normal detector position for low-resolution reflections and one with long exposures and the detector tilted to a 2θ angle of 20°. Reflections were processed using HKL2000 (Otwinowski and Minor, 1997) or Elves (Holton and Alber, 2004) (see Table 1).

### Crystallographic refinement

The hexagonal crystal form was solved by molecular replacement using the RD of diguanylate cyclase (PDB ID 1W25) as the search model. A combination of rigid-body refinement in REFMAC, manual rebuilding in O (Jones *et al*., 1991) and Coot (Emsley and Cowtan, 2004), and simulated annealing in CNS (Brunger *et al*., 1998) improved the structure enough to be completely rebuilt by ARP/wARP (Perrakis *et al*., 1999). Further refinement was performed at 1.3 Å resolution using REFMAC (Murshudov *et al*., 1997). Small increasing-resolution shells were added as anisotropic B-factors, hydrogens, and alternate conformations were added to the model. The final refinement used SHELXL (Sheldrick and Schneider, 1997) with all hydrogens included, and occupancies of alternate conformations were refined with residues in loops grouped together. The model contains 122 residues with 28 alternate conformations and 361 waters.

The WT tetragonal form, the His92Phe mutant and the Tyr102Ala mutant were solved by molecular replacement from the partially refined hexagonal structure of the WT FrzS RD and refined using REFMAC and manual rebuilding in O and Coot. ARP/wARP was used to rebuild the His92Phe and Tyr102Ala structures. This rebuilding process failed for the tetragonal structure due to the poor density for the third monomer. Geometric restraints were tightened to compensate for the disorder of this chain, and as a result, deviations from ideal geometry in the refined, tetragonal structure are lower than average. Molecular graphics were generated with PyMOL (http://pymol.org).

### NMR sample preparation and data collection

*Escherichia coli* BL21 cells harbouring the FrzS RD expression plasmid were grown in terrific broth and exchanged into M9 minimal medium containing 1 g l^−1^ of ^15^N-NH_4_Cl prior to induction with IPTG. Cells were harvested and the wild-type FrzS RD was purified as described above. Prior to NMR experiments, the protein was concentrated to 0.5 mM in a buffer containing 50 mM sodium phosphate, pH 6.3, 50 mM NaCl, 1 mM DTT and 10% ^2^H_2_O. ^15^N-HSQC spectra were collected at 298 °K on a Bruker DRX 600 MHz spectrometer. Data were processed with NMRPipe (Delaglio *et al*., 1995) and analysed with NMRView (Johnson, 2004).

### Strains and growth conditions

*M.xanthus* strains were cultured on CYE rich media (10 mM MOPS pH 7.6, 1% casitone, 0.5% yeast extract and 4 mM MgSO_4_) containing 1.5% agar at 32°C. Liquid cultures were grown in CYE at 32°C with shaking (225 r.p.m.). Standard colony-level social motility assays were performed on CYE containing 0.5% agar. Live cell imaging assays were performed with cells spotted on 0.5 CTT (10 mM Tris pH 7.6, 0.5% Casitone, 1 mM KH_2_PO_4_, 8 mM MgSO_4_) containing 1.5% agar. MMC buffer (10 mM MOPS pH 7.6, 4 mM MgSO_4_, 2 mM CaCl_2_) was used for cell resuspensions. *E. coli* strains were grown in Luria–Bertani media. Kanamycin was used at 100 μg ml^−1^.

### *Myxococcus xanthus* strain construction

In-frame deletions and point mutants in the *frzS* gene were generated by the selection/counter-selection method as previously described (Ueki *et al*., 1996). Briefly, all plasmids used for in-frame deletions were generated by two-step overlap polymerase chain reaction (PCR) extension from *M. xanthus* DZ2 purified chromosomal DNA. One kilobase regions upstream and downstream of the region to be deleted or mutagenized were amplified by an initial round of PCR and fused in a subsequent round by overlap extension. Inserts were cloned into the XbaI and *Hin*dIII sites of the selection/counter-selection plasmid pBJ113. PBJ113 contains a kanamycin resistance gene (*KmR*) and a galactose sensitivity counter-selection gene (*galK*). Deletion and mutagenesis plasmids were electroporated into the relevant strains, and integrants were selected with kanamycin. Plasmids were then looped out via counter-selection with galactose to generate the desired mutation. All strains were verified with a combination of at least two of the following methods: PCR, chromosomal sequencing, Southern blotting, or Western blotting.

### Analysis of motility phenotypes

Colony-level social motility analysis was performed by spotting 4 × 10^7^ cells on nutrient rich CYE media containing 0.5% agar and incubating for 24–48 h at 32°C. Whole-colony images were recorded using a Zeiss dissecting microscope (Model 47 60 09–9901) equipped with a Quantum MP3.3 CCD camera to determine S-motility phenotypes.

### Live cell imaging of FrzS-GFP

Imaging was performed as described previously (Mignot *et al*., 2005). Briefly, cells were harvested and spotted on thin 0.5 CTT 1.5% agar pads on glass slides and covered directly with a glass cover slip. Cells were incubated for 15 min at room temperature in the dark before imaging on a Deltavision fluorescence microscope with an FITC filter set and a 100 × oil-immersion objective (Applied Precision). Cells were filmed for 10 min with 30 s time-lapse. Time-lapse montage images were assembled using Photoshop and Illustrator (Adobe Systems Incorporated).

### Western blotting

Whole cell lysates were prepared from liquid cultures harvested at 4 × 10^8^ cells ml^−1^ and proteins were resolved by SDS-PAGE. Proteins were transferred to nitrocellulose membranes (Bio-Rad) using a tank transfer apparatus (Bio-Rad) in Tris-Glycine buffer containing 20% methanol; membranes were blocked for 30 min in PBS with 0.1% Tween 20 and 5% powdered milk and probed with rabbit anti-FrzS antibody (Mignot *et al*., 2005) at 1:10 000, or mouse anti-GFP monoclonal antibodies (Jackson Immunoresearch) at 1:2000. Anti-mouse or anti-rabbit HRP-conjugated secondary antibodies (Pierce) were used at 1:5000. Immunoreactive complexes were detected by Western Lightning chemiluminescent reagents (NEN) and Kodak Biomax Light film.

### Accession codes

FrzS RD co-ordinates and reflection data have been deposited in the Protein Data Bank under the following IDs: 2GKG (WT hexagonal), 2I6F (WT tetragonal), 2NT3 (Tyr102Ala) and 2NT4 (His92Phe).

## Supplementary material

The following supplementary material is available for this article:

Fig. S1 Phylogenetic tree of sequences with similarity to FrzS RD and KaiA pseudo-RD. The tree shows the average distance by per cent identity. Branches shaded in red represent canonical RDs. The branches shaded in blue contain FrzS-group-RD and related RDs, which are a diverged from canonical RDs. The green branches contain KaiA pseudo-RD and are an outgroup of the canonical RDs. The raw Newick format tree is included in the supplemental material.

Fig. S2 Quantification of FrzS-GFP fluoresence levels of cells shown in Fig. 6. A. Quantification of polar GFP fluoresence in the cell shown in (Fig. 6A). The red line (crosses) indicates the level of GFP fluoresence at the initial lagging cell pole at each time point in the image series. The green line (open boxes) indicates GFP fluoresence levels at the initial leading cell pole. ‘R’ indicates time points at which a reversal took place. Fluoresence levels at all points were normalized to the highest level observed during the time-course (initial leading pole at 1 min). B. Quantification of polar GFP fluoresence in the cell shown in (Fig. 6B). Fluoresence levels at all points were normalized to the highest level observed during the time-course (initial leading pole at 0 min). C. Quantification of polar GFP fluoresence in the cell shown in (Fig. 6C). Fluoresence levels at all points were normalized to the highest level observed during the time-course (initial leading pole at 1 min).

This material is available as part of the online article from:

http://www.blackwell-synergy.com/doi/abs/10.1111/j.1365-2958.2007.05785.x

(This link will take you to the article abstract).
