# Supplementary material for: An atypical receiver domain controls the dynamic polar localization of the Myxococcus xanthus social motility protein FrzS
Source: Mol Microbiol. 2007 Jul 1;65(2):319–32. doi: 10.1111/j.1365-2958.2007.05785.x (PMC1974792; doi:10.1111/j.1365-2958.2007.05785.x)
Supplement: Fig. S1 — Phylogenetic tree of sequences with similarity to FrzS RD and KaiA pseudo-RD. The tree shows the average distance by per cent identity. Branches shaded in red represent canonical RDs. The branches shaded in blue contain FrzS-group-RD and related RDs, which are a diverged from canonical RDs. The green branches contain KaiA pseudo-RD and are an outgroup of the canonical RDs. The raw Newick format tree is included in the supplemental material. [file mmi0065-0319-fs1.pdf]

Fraser_SuppFig1

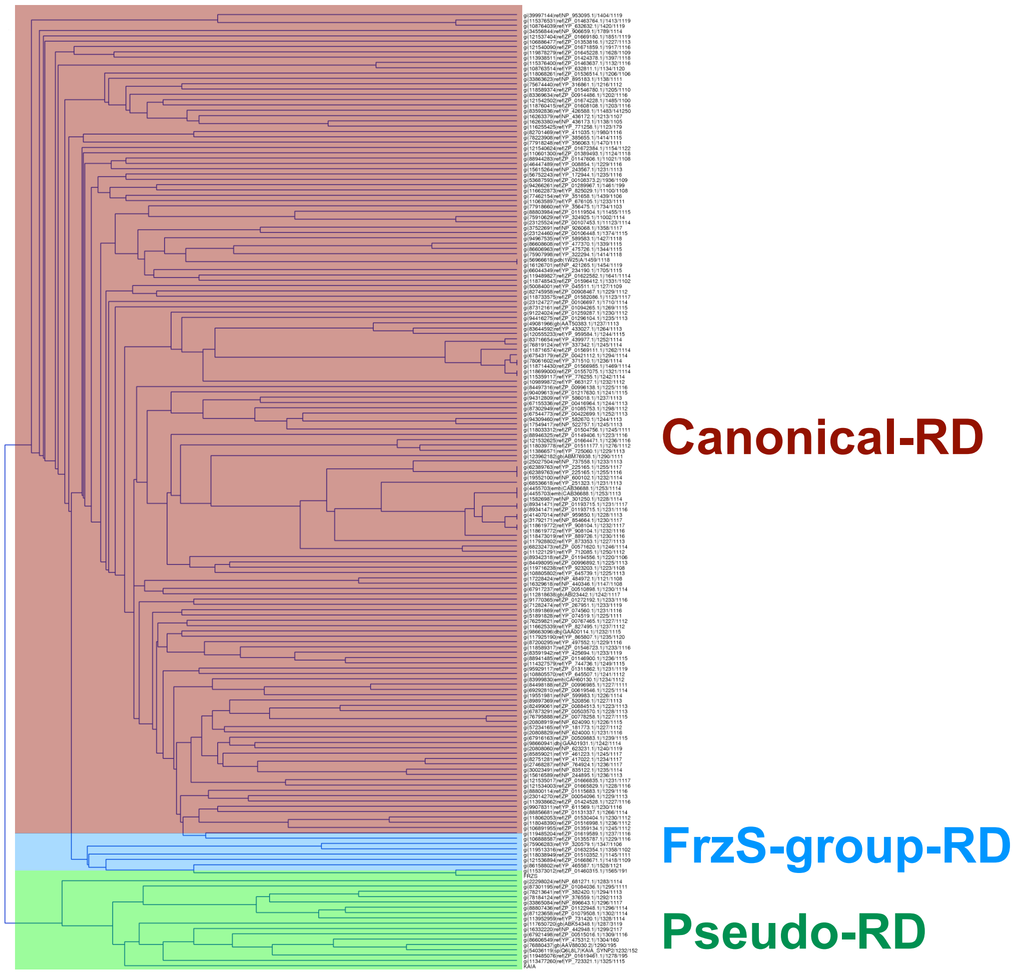

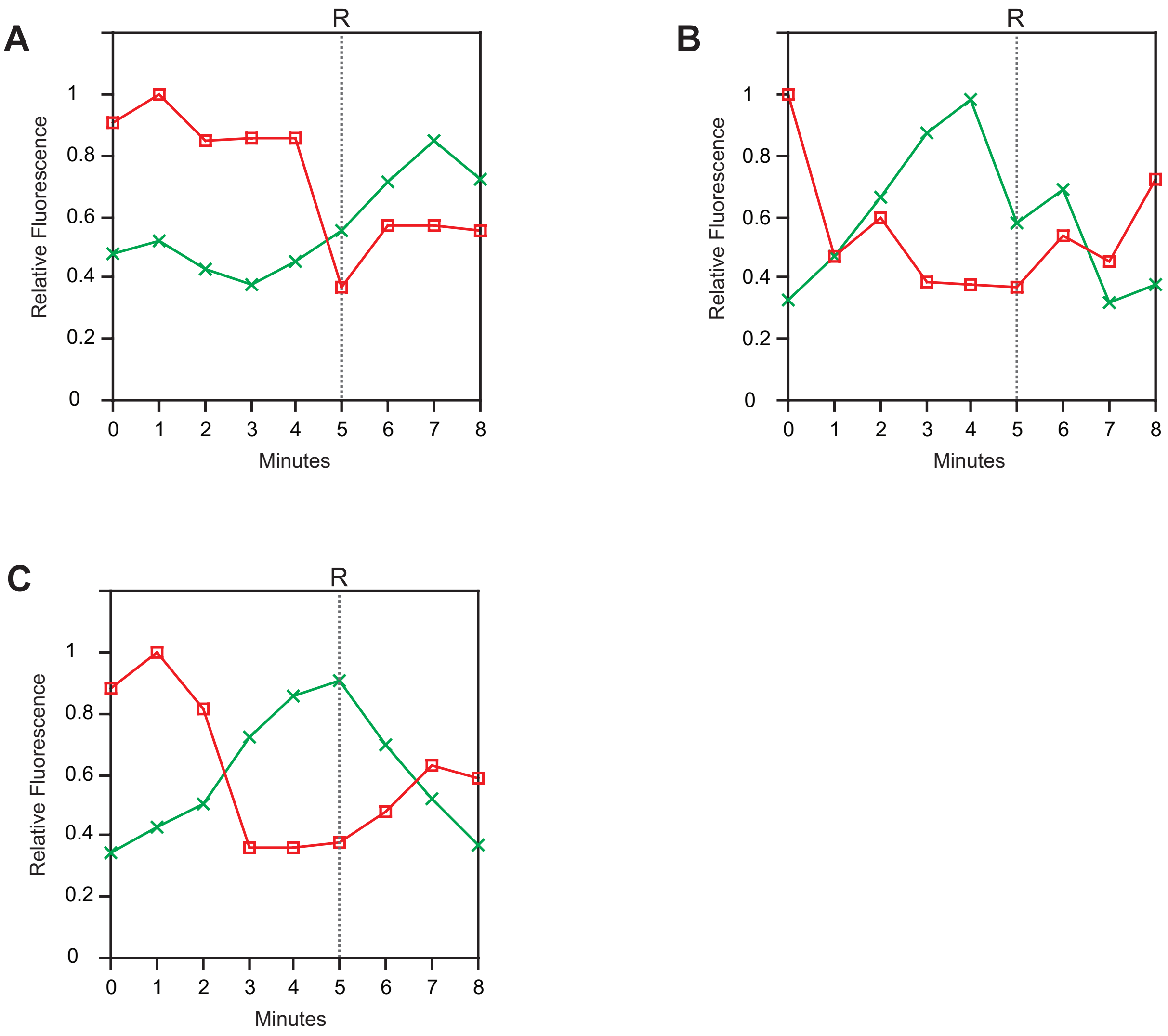
A
R
Relative Fluorescence
1
0.8
0.6
0.4
0.2
0
0 1 2 3 4 5 6 7 8
Minutes
B
R
Relative Fluorescence
1
0.8
0.6
0.4
0.2
0
0 1 2 3 4 5 6 7 8
Minutes
C
R
Relative Fluorescence
1
0.8
0.6
0.4
0.2
0
0 1 2 3 4 5 6 7 8
Minutes

FrzS Tree in Newick Format

(((((gi|115376531|ref|ZP_01463764.1|/1413/1119:3.125,gi|108764039|ref|
YP_632632.1|/1420/1119:3.125):12.1875,gi|39997144|ref|NP_953095.1|/
1404/1119:15.3125):9.670139,(gi|34556844|ref|NP_906659.1|/
1789/1114:24.50786,(gi|121537404|ref|ZP_01669180.1|/1851/1119:24.331701,
(gi|106886477|ref|ZP_01353816.1|/1227/1113:24.217804,((((gi|67547039|ref|
ZP_00424947.1|/1295/1115:0.9375,gi|107022509|ref|YP_620836.1|/
1296/1115:0.9375):17.1875,gi|110835404|ref|YP_694263.1|/1239/1115:18.125):
1.458334,gi|121530027|ref|ZP_01662637.1|/1237/1117:19.583334):4.484989,
((gi|113938511|ref|ZP_01424378.1|/1397/1118:20.625,gi|121536894|ref|
ZP_01668671.1|/1418/1109:20.625):3.3323498,((gi|113934021|ref|
ZP_01419922.1|/1121/1113:19.6875,gi|94968555|ref|YP_590603.1|/
1124/1114:19.6875):4.045582,((gi|115376400|ref|ZP_01463637.1|/
1132/1116:7.1875,gi|108763514|ref|YP_632811.1|/1134/1120:7.1875):16.356855,
((((gi|119878279|ref|ZP_01645228.1|/1628/1109:21.875,gi|121540090|ref|
ZP_01671859.1|/1917/1116:21.875):1.1009083,((gi|78223908|ref|YP_385655.1|/
1414/1115:16.5625,gi|82701469|ref|YP_411035.1|/1980/1116:16.5625):6.124031,
(gi|121540624|ref|ZP_01672384.1|/1154/1122:22.265625,(((gi|56752243|ref|
YP_172944.1|/1235/1116:20.0,gi|53687593|ref|ZP_00108373.2|/1936/1109:20.0):
1.8368912,(((gi|118733575|ref|ZP_01582086.1|/1123/1117:19.6875,(gi|
82745958|ref|ZP_00908467.1|/1229/1112:18.4375,gi|50084001|ref|YP_045511.1|/
1127/1109:18.4375):1.25):1.3391418,(gi|23124727|ref|ZP_00106697.1|/
1710/1114:20.946602,(((gi|67917237|ref|ZP_00510898.1|/1230/1114:18.125,(gi|
17228424|ref|NP_484972.1|/1121/1108:8.125,gi|16329618|ref|NP_440346.1|/
1147/1108:8.125):10.0):1.875,gi|110601300|ref|ZP_01389493.1|/
1124/1118:20.0):0.68418694,(gi|87312161|ref|ZP_01094265.1|/
1269/1115:20.567602,((((((((gi|83644592|ref|YP_433027.1|/
1264/1113:5.3125,gi|120555233|ref|YP_959584.1|/1244/1115:5.3125):
2.03125,gi|49081966|gb|AAT50383.1|/1237/1113:7.34375):8.1119795,((((gi|
118699000|ref|ZP_01557075.1|/1321/1114:0.0,gi|115359117|ref|YP_776255.1|/
1242/1114:0.0):0.625,(gi|67543179|ref|ZP_00421112.1|/1294/1114:0.3125,(gi|
78061602|ref|YP_371510.1|/1236/1114:0.0,gi|118714430|ref|ZP_01566985.1|/
1469/1114:0.0):0.3125):0.3125):1.1875,gi|118716574|ref|ZP_01569111.1|/
1262/1114:1.8125):1.8333333,(gi|83716654|ref|YP_439977.1|/
1252/1114:0.625,gi|76819124|ref|YP_337342.1|/1245/1114:0.625):3.0208333):
11.809896):0.59540653,gi|109899872|ref|YP_663127.1|/1232/1112:16.051136):
1.08428,gi|94416275|ref|ZP_01296104.1|/1235/1113:17.135416):0.604969,gi|
91224024|ref|ZP_01259287.1|/1230/1112:17.740385):1.8641396,((((gi|76259821|
ref|ZP_00767465.1|/1227/1112:16.875,gi|116625339|ref|YP_827495.1|/
1237/1112:16.875):1.4955349,(((((gi|88941485|ref|ZP_01146900.1|/
1236/1115:8.75,gi|114327579|ref|YP_744736.1|/1249/1115:8.75):2.65625,gi|
83591942|ref|YP_425694.1|/1233/1119:11.40625):2.760417,gi|118589317|ref|
ZP_01546723.1|/1233/1116:14.166667):1.848958,gi|87200295|ref|YP_497552.1|/
1229/1116:16.015625):1.921875,(gi|98663096|dbj|GAA00114.1|/
1232/1115:17.1875,gi|117925190|ref|YP_865807.1|/1235/1120:17.1875):0.75):

0.4330349):0.10268021,((((((gi|69292810|ref|ZP_00619546.1|/
1225/1114:7.5,gi|84498188|ref|ZP_00996985.1|/1227/1111:7.5):5.46875,gi|
19551981|ref|NP_599983.1|/1226/1114:12.96875):2.65625,gi|83999830|emb|
CAH60130.1|/1234/1112:15.625):1.9241905,(((gi|121535017|ref|ZP_01666835.1|/
1231/1117:11.875,gi|121534003|ref|ZP_01665829.1|/1228/1116:11.875):4.84375,
(((((gi|30023491|ref|NP_835122.1|/1235/1114:5.625,gi|15616589|ref|
NP_244895.1|/1236/1113:5.625):7.65625,(gi|27468287|ref|NP_764924.1|/
1236/1117:4.6875,gi|82751281|ref|YP_417022.1|/1234/1117:4.6875):8.59375):
1.796875,gi|85859021|ref|YP_461223.1|/1245/1117:15.078125):0.21354198,((gi|
98660941|dbj|GAA01931.1|/1242/1114:13.4375,gi|20808060|ref|NP_623231.1|/
1240/1119:13.4375):1.25,gi|67916163|ref|ZP_00509883.1|/1239/1115:14.6875):
0.604167):0.8523054,((((gi|82499061|ref|ZP_00884513.1|/1223/1113:9.0625,gi|
67873291|ref|ZP_00503570.1|/1228/1113:9.0625):3.59375,(gi|20808919|ref|
NP_624090.1|/1226/1115:1.5625,gi|76795888|ref|ZP_00778258.1|/
1227/1115:1.5625):11.09375):2.03125,gi|89897369|ref|YP_520856.1|/
1227/1113:14.6875):0.90625,(gi|57234165|ref|YP_181773.1|/1227/1112:15.0,gi|
20808829|ref|NP_624000.1|/1231/1116:15.0):0.59375):0.5502224):0.5747776):
0.72610474,(((gi|113938662|ref|ZP_01424528.1|/1227/1116:13.75,gi|23014270|
ref|ZP_00054096.1|/1229/1113:13.75):2.03125,gi|88800114|ref|ZP_01115683.1|/
1229/1116:15.78125):1.4508934,((gi|119485204|ref|ZP_01619589.1|/
1237/1116:15.9375,gi|106888587|ref|ZP_01355787.1|/1229/1116:15.9375):
1.1875,(((gi|118062053|ref|ZP_01530404.1|/1230/1112:6.5625,gi|118048390|
ref|ZP_01516998.1|/1236/1112:6.5625):5.15625,gi|106891955|ref|
ZP_01359134.1|/1245/1112:11.71875):4.583334,(gi|99078311|ref|YP_611569.1|/
1230/1116:15.3125,gi|88856681|ref|ZP_01131337.1|/1266/1114:15.3125):
0.98958397):0.82291603):0.1071434):0.21271133):0.104335785):0.5556488,(gi|
95929117|ref|ZP_01311862.1|/1231/1119:16.25,gi|108805570|ref|YP_645507.1|/
1241/1112:16.25):1.8548393):0.21428871,(gi|51891828|ref|YP_074519.1|/
1225/1111:17.1875,gi|90409613|ref|ZP_01217630.1|/1241/1115:17.1875):
1.131628):0.15408707):0.8485184,(gi|71282474|ref|YP_267951.1|/
1233/1119:16.5625,gi|91770365|ref|ZP_01272192.1|/1233/1116:16.5625):
2.7592335):0.28279114):0.24525642,(gi|84497316|ref|ZP_00996138.1|/
1225/1116:19.527029,(((gi|112818638|gb|ABI23442.1|/1242/1117:18.599539,
((((((((gi|62389763|ref|YP_225165.1|/1255/1117:0.0,(gi|19552100|ref|
NP_600102.1|/1232/1114:0.0,gi|62389763|ref|YP_225165.1|/1255/1116:0.0):
0.0):5.3125,gi|25027504|ref|NP_737558.1|/1233/1113:5.3125):4.420573,
((((((gi|118619772|ref|YP_908104.1|/1232/1117:0.0,gi|118619772|ref|
YP_908104.1|/1232/1116:0.0):0.625,(gi|31792171|ref|NP_854664.1|/
1230/1117:0.0,gi|41407014|ref|NP_959850.1|/1228/1113:0.0):0.625):1.171875,
(gi|89341471|ref|ZP_01193715.1|/1231/1117:0.0,gi|89341471|ref|
ZP_01193715.1|/1231/1116:0.0):1.796875):0.390625,(gi|4455703|emb|
CAB36688.1|/1253/1114:0.0,(gi|15826987|ref|NP_301250.1|/1228/1114:0.0,gi|
4455703|emb|CAB36688.1|/1253/1113:0.0):0.0):2.1875):0.48611116,gi|
118473019|ref|YP_889726.1|/1230/1116:2.6736112):4.263889,gi|68536618|ref|
YP_251323.1|/1231/1113:6.9375):2.409091,gi|117928802|ref|YP_873353.1|/
1227/1113:9.346591):0.38648224):1.3509111,(gi|68232473|ref|ZP_00571620.1|/

1246/1114:2.8125,gi|111221291|ref|YP_712085.1|/1250/1112:2.8125):8.271484):
4.6104603,gi|123962182|gb|ABM76938.1|/1290/1111:15.694445):1.5369148,(gi|
121532625|ref|ZP_01664471.1|/1236/1116:6.09375,(gi|113866571|ref|
YP_725060.1|/1229/1113:5.0,gi|118039778|ref|ZP_01511177.1|/1276/1112:5.0):
1.09375):11.1376095):0.45330048,gi|88946325|ref|ZP_01149406.1|/
1223/1116:17.68466):0.26371002,(((gi|84498095|ref|ZP_00996892.1|/
1225/1113:14.6875,gi|119716238|ref|YP_923203.1|/1223/1108:14.6875):
0.78125,gi|108805802|ref|YP_645739.1|/1225/1113:15.46875):1.302084,gi|
89342318|ref|ZP_01194556.1|/1220/1106:16.770834):1.177536):0.6511688):
0.2206173,((gi|94312809|ref|YP_586018.1|/1237/1113:13.125,gi|67155336|ref|
ZP_00416964.1|/1244/1113:13.125):2.9375,(gi|87302949|ref|ZP_01085753.1|/
1298/1112:13.4375,((gi|67544773|ref|ZP_00422699.1|/1252/1113:10.9375,(gi|
94309460|ref|YP_582670.1|/1244/1113:3.125,gi|17549417|ref|NP_522757.1|/
1245/1113:3.125):7.8125):0.833333,gi|118033312|ref|ZP_01504756.1|/
1245/1111:11.770833):1.666667):2.625):2.757656):0.3941307,(gi|51891869|ref|
YP_074560.1|/1231/1116:18.75,gi|77918660|ref|YP_356475.1|/1734/1103:18.75):
0.4642868):0.31274223):0.322752):0.7178211):0.11658478):0.26241493):
0.08003998):0.44258118,(((((((gi|86606963|ref|YP_475726.1|/
1344/1115:4.375,gi|86608608|ref|YP_477370.1|/1339/1115:4.375):10.15625,gi|
75907998|ref|YP_322294.1|/1414/1118:14.53125):2.447916,(gi|56966618|pdb|
1W25|A/1459/1118:0.0,gi|16126701|ref|NP_421265.1|/1454/1119:0.0):
16.979166):1.083334,gi|94967535|ref|YP_589583.1|/1427/1118:18.0625):
1.0173626,((gi|119489827|ref|ZP_01622582.1|/1641/1114:16.25,gi|118748543|
ref|ZP_01596412.1|/1331/1102:16.25):2.34375,gi|66044349|ref|YP_234190.1|/
1705/1115:18.59375):0.4861126):0.36458206,(gi|23124460|ref|ZP_00106448.1|/
1374/1115:13.125,gi|37522691|ref|NP_926068.1|/1358/1117:13.125):6.3194447):
1.7260075,(((gi|75910629|ref|YP_324925.1|/11002/1114:3.125,gi|23125524|ref|
ZP_00107453.1|/11123/1114:3.125):8.28125,gi|88803984|ref|ZP_01119504.1|/
11455/1115:11.40625):9.114582,(gi|94266261|ref|ZP_01289967.1|/
1461/199:18.4375,gi|116622873|ref|YP_825029.1|/11100/1108:18.4375):
2.083332):0.64962006):0.2987709):0.36766815):0.29810905,(gi|15615264|ref|
NP_243567.1|/1231/1113:20.78125,(gi|46447489|ref|YP_008854.1|/
1229/1116:20.0,gi|88944283|ref|ZP_01147606.1|/11021/1108:20.0):0.78125):
1.3537502):0.13062477):0.42090607):0.2893772):0.05070305,(((gi|119513316|
ref|ZP_01632354.1|/1358/1102:10.0,gi|75906283|ref|YP_320579.1|/
1347/1106:10.0):12.03125,gi|118038949|ref|ZP_01510352.1|/
1145/1111:22.03125):0.51041603,(((gi|115373012|ref|ZP_01460315.1|/
1565/191:3.125,(FRZS:0.0,gi|108759929|ref|YP_632324.1|/1562/1119:0.0):
3.125):14.166666,gi|86158802|ref|YP_465587.1|/1528/1121:17.291666):
4.427084,gi|77918248|ref|YP_356063.1|/1470/1111:21.71875):0.82291603):
0.4849453):0.06435394,(((gi|110635897|ref|YP_676105.1|/1233/1111:20.0,gi|
77462154|ref|YP_351658.1|/1439/1106:20.0):1.2073841,(gi|118068261|ref|
ZP_01536514.1|/1206/1106:21.09375,(gi|33863623|ref|NP_895183.1|/
1138/1111:20.902779,((gi|83369634|ref|ZP_00914486.1|/1202/1116:19.791666,
((gi|121542502|ref|ZP_01674228.1|/1485/1100:18.125,gi|118760415|ref|
ZP_01608108.1|/1203/1116:18.125):0.78125,(gi|83592836|ref|YP_426588.1|/

11483/141250:16.875,((gi|116255425|ref|YP_771258.1|/1123/179:9.6875,gi|
16263380|ref|NP_436173.1|/1138/1105:9.6875):6.5625,gi|16263379|ref|
NP_436172.1|/1213/1107:16.25):0.625):2.03125):0.88541603):0.25297737,(gi|
118589374|ref|ZP_01546780.1|/1205/1110:17.1875,gi|75674440|ref|
YP_316861.1|/1216/1112:17.1875):2.8571434):0.8581352):0.19097137):
0.11363411):0.59550095,gi|75675220|ref|YP_317641.1|/1135/1103:21.802885):
1.2880802):0.45339012):0.18872643):0.22426796):0.11097336):0.14948082):
0.11389732):0.1761589):0.47477913):1.459629,(gi|22298024|ref|NP_681271.1|/
1283/1114:23.385416,(((((gi|78213641|ref|YP_382420.1|/1294/1113:8.125,gi|
78184124|ref|YP_376559.1|/1292/1113:8.125):4.53125,gi|33865084|ref|
NP_896643.1|/1296/1117:12.65625):0.7118063,(gi|113952959|ref|YP_731420.1|/
1328/1114:10.15625,(gi|87123658|ref|ZP_01079508.1|/1302/1114:8.75,gi|
88807436|ref|ZP_01122948.1|/1296/1114:8.75):1.40625):3.2118063):
5.7986097,gi|87301195|ref|ZP_01084036.1|/1295/1111:19.166666):1.8763542,
((KAIA:0.0,(gi|49258679|pdb|1R8J|A/1289/3119:0.0,gi|56750341|ref|
YP_171042.1|/1284/3119:0.0):0.0):20.117188,(gi|117650720|gb|ABK54348.1|/
1287/3119:19.375,((gi|16332220|ref|NP_442948.1|/1299/2117:16.5625,(gi|
67921498|ref|ZP_00515016.1|/1309/1116:14.609375,((gi|86606549|ref|
YP_475312.1|/1304/160:9.375,(gi|76880437|gb|AAV88030.2|/1290/195:5.625,gi|
54036119|sp|Q6L8L7|KAIA_SYNP2/1232/152:5.625):3.75):3.125,gi|119485076|ref|
ZP_01619461.1|/1278/195:12.5):2.109375):1.953125):1.5625,gi|113477260|ref|
YP_723321.1|/1325/1115:18.125):1.25):0.7421875):0.92583275):2.3423958):
3.0568523):0.63503456,gi|108763235|ref|YP_631200.1|/11395/1121:27.077303);
